# Varus deformity in medial knee osteoarthritis correlates with fatty degeneration of lower limb muscles: Artificial intelligence‐based computed tomography analysis

**DOI:** 10.1002/jeo2.70518

**Published:** 2025-11-14

**Authors:** Minami Suzuki, Tomofumi Kinoshita, Kohei Kono, Mazen Soufi, Yoshito Otake, Keisuke Uemura, Tatsuhiko Kutsuna, Kazunori Hino, Yoko Murakami, Yoshiyuki Watanabe, Yoshinobu Sato, Masaki Takao

**Affiliations:** ^1^ Department of Orthopaedic Surgery Ehime University Graduate School of Medicine Toon Ehime Japan; ^2^ Division of Information Science, Graduate School of Science and Technology Nara Institute of Science and Technology Ikoma Nara Japan; ^3^ Department of Orthopaedic Medical Engineering, Graduate School of Medicine Osaka University Suita Osaka Japan; ^4^ Department of Radiology Shiga University of Medical Science Otsu Shiga Japan

**Keywords:** artificial Intelligence, hip–knee–ankle angle, knee osteoarthritis, muscle fatty degeneration, muscle mass

## Abstract

**Purpose:**

As knee osteoarthritis (KOA) progresses, lower limb alignment deteriorates, severely impairing daily activities. However, the impact of lower limb malalignment on muscle atrophy and degeneration from the trunk to the foot remains unclear. This study aimed to elucidate the effects of varus deformity on muscle volume and fatty degeneration from the trunk to the foot in medial KOA.

**Methods:**

This study included 79 patients with end‐stage medial KOA (16 males and 63 females). The hip–knee–ankle (HKA) angle, measured on standing leg radiographs, served as an index of varus deformity. Ten muscle groups were segmented on computed tomography (CT) images using an artificial intelligence‐based method. Muscle volume was standardised by dividing by height squared, and the mean CT value for each muscle group was calculated as an index of fatty degeneration. Multiple linear regression analysis was used to assess the correlation between muscle volume or CT value and HKA, adjusting for age, sex and body mass index. Statistical significance was set at *p* < 0.05.

**Results:**

The mean HKA was 10.2° (standard deviation 5.9°, range 0–30°). Multiple regression analysis showed a significant association between HKA and the mean CT values of the gluteus maximus, gluteus medius and minimus, adductor muscles, quadriceps and deep and superficial posterior compartments of the lower leg (*β* = −0.50, *p* < 0.001; *β* = −0.25, *p* = 0.048; *β* = −0.32, *p* = 0.007; *β* = −0.39, *p* < 0.001; *β* = −0.38, *p* = 0.001; and *β* = −0.42, *p* < 0.001, respectively).

**Conclusion:**

Varus deformity in medial KOA correlates with fatty degeneration of lower limb muscles rather than muscle volume. KOA progression affects not only the muscles around the knee but also those in the trunk, hip and lower leg.

**Level of Evidence:**

Level III.

**Abbreviations**
2D: two‐dimensional
3D: three‐dimensional
CT: computed tomography
HKA: hip–knee–ankle
HU: Hounsfield unit
KOA: knee osteoarthritis
MRI: magnetic resonance imaging

## INTRODUCTION

Knee osteoarthritis (KOA) causes chronic knee pain and functional impairment of the musculoskeletal system as the disease progresses. Medial compartment osteoarthritis is the most common type, accounting for 60%–70% of all cases [38, 45]. Deformity in limb alignment, measured as the hip–knee–ankle (HKA) angle, is common in patients with KOA. An increase in HKA (varus deformity) is observed with the progression of medial KOA [23, 38]. Varus deformity in lower limb alignment generates localised mechanical stress on the medial tibiofemoral joint during weight‐bearing, accelerating cartilage wear [41], which exacerbates inflammation and knee pain and impairs physical activity and quality of life [32].

Impaired physical activity and quality of life significantly correlate with muscle atrophy and degeneration from the trunk to the foot [19]. However, the relationship between the progression of varus deformity in limb alignment and muscle condition remains inadequately investigated. Quadriceps muscle strength has a significant impact on KOA progression [24, 26, 42]. A comprehensive understanding of muscle conditions throughout the trunk to the foot may provide deeper insights into the pathophysiology of KOA.

Previous research has primarily relied on two‐dimensional (2D) and qualitative methods, such as computed tomography (CT) and magnetic resonance imaging (MRI), for muscle evaluation [24, 28, 36]. Advances in three‐dimensional (3D) imaging techniques provide more comprehensive information than 2D methods [36]. Recent deep‐learning developments have enabled the automatic segmentation of muscle groups, allowing precise 3D analysis of individual muscles using CT or MRI images [15, 19, 22, 43].

This study aimed to elucidate the effects of varus deformity on muscle volume and fatty degeneration from the trunk to the foot in medial KOA. Furthermore, the muscle volume and CT values of individual muscle groups were analysed using a deep‐learning model in patients with end‐stage KOA.

## METHODS

Of 364 patients with KOA who underwent total knee arthroplasty at our institution between April 2021 and March 2024, 101 agreed to participate and completed a questionnaire. After applying the exclusion criteria described below, 79 patients were included in this cross‐sectional study. This study was approved by the institutional review board of our institution (IRB number 2103009). The exclusion criteria were: (1) lack of preoperative CT images, (2) a history of trauma, infection, tumour or osteoarthritis other than the knee in the affected limb and (3) valgus deformity. Patient characteristics are shown in Table 1. All knees were classified as Kellgren–Lawrence grade 4 [20]. The HKA angle, a measurement of lower limb alignment, was obtained from a full‐length, weight‐bearing anteroposterior radiograph of the affected lower limb [30]. In this study, the HKA angle was measured once per case by the first author and another board‐certified orthopaedic surgeon. Inter‐rater reliability for the HKA angle measurement was excellent, with an ICC (two‐way mixed‐effects model, absolute agreement) of 0.990 (95% confidence interval [CI]: 0.985–0.994) for single measurements and 0.995 (95% CI: 0.992–0.997) for average measurements.

**Table 1:** Patient characteristics.

	Total mean ± SD (range)
Number of patients	63 females, 16 males
Age (years)	76.3 ± 6.7 (57–89)
Body mass index (kg/m^2^)	25.5 ± 3.9 (18.7–36.9)
HKA angle (°)	10.2 ± 5.9 (0–30)
Maximum knee extension angle (°)	−9.9 ± 8.3 (−30 to 0)
Maximum knee flexion angle (°)	120.9 ± 15.8 (65–145)

Abbreviations: HKA, hip–knee–ankle; SD, standard deviation.

CT images were acquired for preoperative planning using a 320‐row multidetector CT system (Aquilion ONE GENESIS Edition, Canon Medical Systems Inc.) with the following protocol: tube voltage, 120 kV; tube current, 50–520 mA; helical pitch, 65.0; slice thickness, 1.00 mm; and X‐ray tube rotation speed, 0.5 s. The imaging range extended from the 10th thoracic vertebra to the toes.

**Figure 1:**
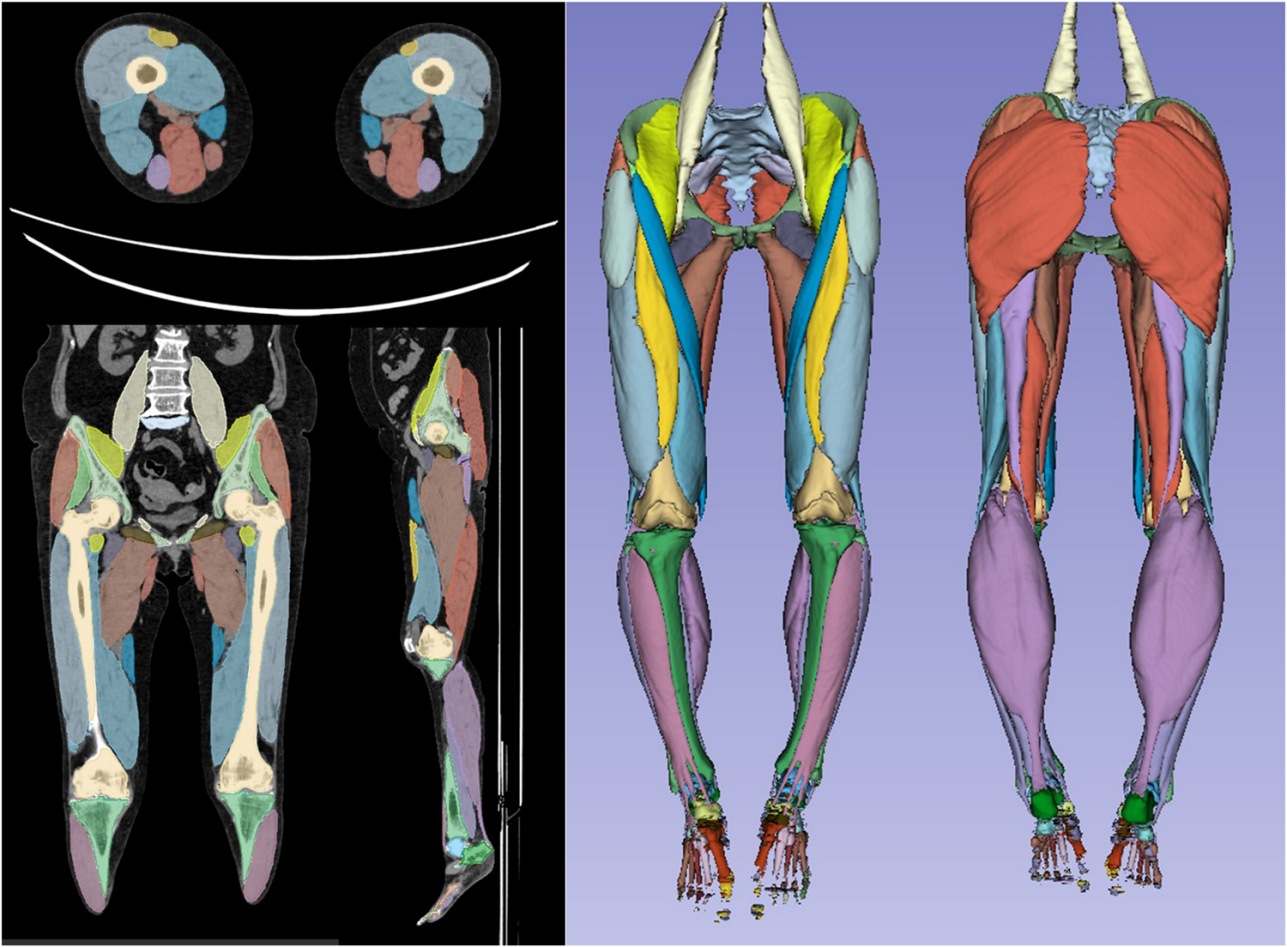
Artificial intelligence‐based segmentation model of a patient with knee osteoarthritis. The left image shows the precise segmentation of each muscle group. The right image presents a three‐dimensional skeletal muscle model reconstructed from segmentation images.

A deep‐learning‐based AI model, built on a fully convolutional neural network with a U‐Net architecture (Bayesian U‐Net), which has high reliability in bone and muscle assessment in hip‐to‐knee CTs [15], was used in this study. Muscles were divided into 10 groups according to their function: gluteus maximus, gluteus medius and minimus, iliopsoas, adductors, quadriceps, hamstrings, anterior compartment, lateral compartment, deep posterior compartment and superficial posterior compartment muscles of the lower leg. AI segmentation models were trained and validated on the CT images used in this study. Two researchers generated ground‐truth labels for target muscles in a subset of 34 cases using an active learning approach [15]. A cross‐validation experiment was conducted to assess the accuracy of the AI models. To evaluate muscle mass, each muscle volume was divided by height squared (cm^3^/m^2^) and defined as the standardised muscle volume. The average CT value (Hounsfield unit [HU]) for each segmented muscle group was calculated as an index of fatty muscle degeneration (Figure 1). Attenuation of CT values is reportedly associated with skeletal muscle lipid content [14].

### Statistical analysis

Normality was assessed using the Shapiro–Wilk test. The CT values for each muscle and HKA subscale were not normally distributed; however, muscle volumes showed a normal distribution. Bivariate analysis was conducted between HKA subscales and standardised muscle volume or mean CT values using Spearman's rank correlation coefficient tests. Multiple linear regression analysis was performed using the muscle groups that showed statistically significant correlations with HKA as independent variables. Confounding factors, including age, sex and body mass index, were included as adjustment variables. Statistical significance was set at *p* < 0.05. All statistical analyses were performed using SPSS (version 28.0, IBM Japan).

### AI model validation

The segmentation accuracy (Dice coefficient) of the muscles in the cross‐validation experiment (34 cases) using the AI model and the average surface error were 0.958 ± 0.020 and 0.560 ± 0.770 mm, respectively. The accuracy of muscle volume and mean HU measurements was 2.690 ± 2.870% and 0.647 ± 0.707 HU, respectively [22].

## RESULTS

### Muscle measurements

The standardised muscle volume and mean CT value of each muscle group in the affected side of patients with KOA are presented in Table 2. HKA was not significantly associated with any standardised muscle volume in either the univariate analysis (Table 3) or the multiple linear regression analysis. In contrast, significant correlations were observed between HKA and the mean CT values of all examined muscles in the univariate analysis (Table 3). In the multiple linear regression analysis, the mean CT values of the gluteus maximus, gluteus medius and minimus, adductor muscles, quadriceps and deep and superficial posterior compartments of the lower leg remained significantly correlated with HKA (Table 4).

**Table 2:** Muscle atrophy and degree of fatty degeneration on the affected side were assessed using standardised muscle volume and CT values.

	Standardised muscle volume (cm^3^/m^2^)	CT values (HU)
	Mean ± SD	Mean ± SD
Gluteus maximus	273.0 ± 59.4	16.8 ± 11.9
Gluteus medius and minimus	125.5 ± 17.5	26.1 ± 10.2
Iliopsoas	41.0 ± 6.9	48.8 ± 4.3
Adductor muscles	279.1 ± 54.2	35.2 ± 6.1
Quadriceps	351.8 ± 76.5	40.6 ± 7.0
Hamstrings	198.2 ± 39.5	30.1 ± 9.2
Anterior compartment	76.8 ± 13.8	41.8 ± 6.4
Lateral compartment	39.2 ± 8.9	43.3 ± 6.6
Deep posterior compartment	90.0 ± 16.9	37.5 ± 7.4
Superficial posterior compartment	220.1 ± 53.1	34.6 ± 9.5

Abbreviations: CT, computed tomography; HU, Hounsfield unit; SD, standard deviation.

**Table 3:** Correlation between HKA angle and standardised muscle volume or CT values.

	Standardised muscle volume (cm^3^/m^2^)		CT values (HU)	
	*ρ*	*p*	*ρ*	*p*
Gluteus maximus	−0.048	0.674	−0.492	<0.001**
Gluteus medius and minimus	−0.140	0.219	−0.457	<0.001**
Iliopsoas	−0.066	0.561	−0.338	0.002**
Adductor muscles	0.047	0.684	−0.466	<0.001**
Quadriceps	−0.073	0.523	−0.526	<0.001**
Hamstrings	−0.134	0.238	−0.349	0.002**
Anterior compartment	−0.180	0.112	−0.333	0.003**
Lateral compartment	−0.085	0.455	−0.385	<0.001**
Deep posterior compartment	−0.008	0.947	−0.504	<0.001**
Superficial posterior compartment	−0.021	0.855	−0.505	<0.001**

Abbreviations: CT, computed tomography; HKA, hip–knee–ankle; HU, Hounsfield unit.

** *p* < 0.01.

**Table 4:** Multiple linear regression analysis to evaluate the correlation between HKA angle and standardised muscle volume or CT values.

	*β*	95% CI	*p*
CT values			
Gluteus maximus	−0.50	−0.37 to −0.12	<0.001**
Gluteus medius and minimus	−0.25	−0.29 to −0.002	0.048*
Adductor muscles	−0.32	−0.53 to −0.09	0.007**
Quadriceps	−0.39	−0.50 to −0.13	<0.001**
Deep posterior compartment	−0.38	−0.50 to −0.12	0.001**
Superficial posterior compartment	−0.42	−0.40 to −0.12	<0.001**

Abbreviations: CI, confidence interval; CT, computed tomography; HKA, hip–knee–ankle; β, standard regression coefficient.

* *p* < 0.05

** *p* < 0.01.

## DISCUSSION

This study used a deep learning model to automatically segment muscle groups from lower limb CT images, and the relationship between fatty degeneration in each muscle group and lower limb alignment was examined. The results demonstrated that the degree of varus alignment in KOA is associated with fatty degeneration in the gluteus maximus, gluteus medius and minimus, adductor muscles, quadriceps and deep and superficial posterior compartments of the lower leg. Notably, despite focusing on patients with severe KOA, the correlation between limb alignment and muscle condition extended beyond the knee to include the hip and lower leg muscles.

This study is the first to demonstrate that a greater number of muscle groups exhibited a correlation between lower limb alignment and fat degeneration compared to muscle volume. Previous studies have reported that muscle strength and physical performance depend more on muscle density than on muscle volume [14, 44]. Although many studies have investigated fatty degeneration in the quadriceps of patients with medial KOA by comparing them to individuals without KOA [24, 28], few have evaluated fatty degeneration in the trunk, hip and lower leg muscles despite the severe impairment in daily living among patients with KOA. The novelty of our study lies in its application of AI‐based analysis, which allowed for the simultaneous quantification of fatty degeneration from the trunk to the foot, thereby underscoring the importance of evaluating this condition beyond the quadriceps.

Previous studies have consistently reported reduced hip muscle strength in patients with KOA compared with healthy controls [10, 16, 18]. Besides exercises targeting the muscles surrounding the knee, strengthening the hip muscles has been shown to contribute to symptom improvement [4, 5, 7, 17, 37]. In this study, Fatty degeneration of the gluteus maximus showed a significant correlation with varus alignment. The gluteus maximus primarily functions in hip extension [35] and reaches peak activity during heel contact in normal gait [46]. It also plays a crucial role in generating the knee extension moment during the stance phase [1]. However, patients with medial KOA tend to exhibit reduced knee flexion during stance [12] and decreased external knee extension moment [33]. These gait characteristics likely reduce the contribution of the gluteus maximus to knee extension, potentially contributing to its fatty degeneration. The gait characteristics of medial KOA may, therefore, influence the fatty degeneration of the gluteus maximus.

The gluteus medius and minimus play a crucial role in maintaining pelvic stability [21, 40]. In patients with medial KOA, weakness of the gluteus medius and minimus can lead to contralateral pelvic drops during gait. This postural change alters the centre of mass, thereby accelerating disease progression [7, 31]. Notably, strengthening of the gluteus medius and minimus has been reported to reduce the risk of KOA progression [7, 8]. The present finding that the degree of varus deformity is associated with the severity of fatty degeneration in the gluteus medius and minimus supports this chain of biomechanical consequences.

Although several studies have reported that patients with KOA present with reduced hip adductor strength compared to healthy controls [10, 16, 37], the relationship between lower limb alignment and the adductor condition has not been previously examined. To our knowledge, this is the first study to demonstrate that greater degrees of varus deformity are significantly associated with more pronounced fatty degeneration in the hip adductor muscles. One possible explanation is that, during gait, the hip adductor muscles contribute to hip extension [9]; however, patients with medial KOA often exhibit a reduced range of hip extension [2, 39], which may, in turn, limit the activation and loading of these muscles.

Fatty degeneration of the quadriceps was also associated with varus deformity progression. Varus deformity in KOA is often accompanied by varus thrust during gait [6, 25], which increases the load on the medial knee compartment and contributes to osteoarthritis progression. A lower isokinetic knee extensor muscle strength has been linked to greater varus thrust magnitude in individuals with KOA [11]. These findings suggest that interventions targeting quadriceps fatty degeneration may be beneficial, even in patients with severe varus deformity.

The primary muscles in the posterior compartment of the lower leg, the gastrocnemius and soleus, exhibit peak activity during the late stance phase of gait, contributing to ankle plantarflexion and knee flexion to support toe‐off [27, 34, 46]. Neptune et al. revealed that the gastrocnemius and soleus muscles contribute to vertical trunk acceleration during the stance phase [34]. However, patients with medial KOA often demonstrate increased knee flexion during late stance. Ghazwan et al. reported reduced gastrocnemius activity during gait in patients with severe KOA compared with controls [13]. This diminished contraction during late stance may explain the observed correlation between fatty degeneration of the posterior lower leg muscles and varus deformity.

To our knowledge, no longitudinal studies have investigated the relationship between muscle characteristics and alignment changes in medial KOA, leaving it unclear whether muscle degeneration is a cause or a consequence of malalignment. Patients with KOA often adopt altered gait patterns due to pain and malalignment [3], which can lead to imbalanced muscle activation. This disuse and subsequent physical inactivity are known contributors to muscle fatty degeneration [29]. Therefore, it is plausible that this mechanical imbalance drives the degeneration observed in certain muscles. The resulting decline in muscle function may, in turn, exacerbate joint instability and further progress the deformity, creating a vicious cycle. Based on these previous findings together with the present results, we consider that the mechanical stress from a varus deformity induces disuse of specific muscle groups, thereby promoting muscle fatty degeneration. Our findings suggest that targeted rehabilitation for the muscle groups associated with varus deformity, as identified in this study, could help interrupt this cycle and improve patient outcomes. This approach may contribute to the development of early intervention and comprehensive rehabilitation strategies. However, the causal relationship between the progression of lower limb deformity and changes in muscle fatty degeneration needs to be further examined with a larger sample size.

This study had limitations. First, as noted above, the sample size was small (79 patients, including 16 males), and sex differences were not analysed. Future research should focus on sex‐specific differences in muscle mass and fatty degeneration in patients with KOA. Further studies with larger sample sizes are required to validate these findings. Second, the study did not include a control group. To understand the effects of KOA on muscle atrophy and fatty degeneration better, comparisons with age‐ and sex‐matched healthy controls are essential. Another limitation of this study is that the analysis focused solely on the relationship between fatty degeneration and volume of the muscles on the affected side and varus deformity of the lower limb. In KOA, bilateral knee involvement is not uncommon, and the condition of the contralateral side may also influence the results. Furthermore, in the present cohort, the full‐length alignment of the contralateral limb was not consistently assessed across all cases; therefore, further investigation is warranted.

## CONCLUSION

Lower limb alignment in KOA is correlated with fatty degeneration of the gluteus maximus, gluteus medius and minimus, adductor muscles, quadriceps and deep and superficial posterior compartments of the lower leg. The progression of varus deformity in KOA affects not only symptomatic muscle groups around the knee joint but also proximal hip muscles and lower leg muscle groups. However, to expand upon these findings, future studies should employ larger sample sizes and include comparisons with age‐ and sex‐matched healthy controls.

## AUTHOR CONTRIBUTIONS

**Minami Suzuki:** Writing—original draft. **Tomofumi Kinoshita:** Conceptualisation; writing—review and editing. **Kohei Kono:** Formal analysis, investigation. **Mazen Soufi:** Methodology; formal analysis; investigation; funding acquisition. **Yoshito Otake:** Methodology; funding acquisition. **Keisuke Uemura:** Methodology. **Tatsuhiko Kutsuna:** Writing—review and editing. **Kazunori Hino:** Methodology. **Yoko Murakami:** Methodology. **Yoshiyuki Watanabe:** Methodology. **Yoshinobu Sato:** Funding acquisition; resources. **Masaki Takao:** Conceptualisation; methodology; writing—review and editing; resources; supervision.

## CONFLICT OF INTEREST STATEMENT

The authors declare no conflicts of interest.

## ETHICS STATEMENT

This study was approved by the Institutional Review Board of Ehime University (identification number: 2103009). All participants provided written informed consent.

## Data Availability

The data sets generated and/or analysed during the current study are available from the corresponding author on reasonable request.
